# Examining the possible causal relationship between lung function, COPD and Alzheimer’s disease: a Mendelian randomisation study

**DOI:** 10.1136/bmjresp-2020-000759

**Published:** 2021-07-07

**Authors:** Daniel Higbee, Raquel Granell, Esther Walton, Roxanna Korologou-Linden, George Davey Smith, James Dodd

**Affiliations:** 1 Academic Respiratory Unit, Southmead Hospital, Bristol, UK; 2 MRC Integrative Epidemiology Unit, University of Bristol, Bristol, UK; 3 Department of Psychology, University of Bath, Bath, Somerset, UK

**Keywords:** COPD epidemiology, lung physiology

## Abstract

**Rationale:**

Large retrospective case-control studies have reported an association between chronic obstructive pulmonary disease (COPD), reduced lung function and an increased risk of Alzheimer’s disease. However, it remains unclear if these diseases are causally linked, or due to shared risk factors. Conventional observational epidemiology suffers from unmeasured confounding and reverse causation. Additional analyses addressing causality are required.

**Objectives:**

To examine a causal relationship between COPD, lung function and Alzheimer’s disease.

**Methods:**

Using two-sample Mendelian randomisation, we used single nucleotide polymorphisms (SNPs) identified in a genome wide association study (GWAS) for lung function as instrumental variables (exposure). Additionally, we used SNPs discovered in a GWAS for COPD in those with moderate to very severe obstruction. The effect of these SNPs on Alzheimer’s disease (outcome) was taken from a GWAS based on a sample of 24 807 patients and 55 058 controls.

**Results:**

We found minimal evidence for an effect of either lung function (OR: 1.02 per SD; 95% CI 0.91 to 1.13; p value 0.68) or liability for COPD on Alzheimer’s disease (OR: 0.97 per SD; 95% CI 0.92 to 1.03; p value 0.40).

**Conclusion:**

Neither reduced lung function nor liability COPD are likely to be causally associated with an increased risk of Alzheimer’s, any observed association is likely due to unmeasured confounding. Scientific attention and health prevention policy may be better focused on overlapping risk factors, rather than attempts to reduce risk of Alzheimer’s disease by targeting impaired lung function or COPD directly.

**Key messages**

**What is the key question?**

- Is their evidence that reduced lung function and chronic obstructive pulmonary disease (COPD) cause an increased risk of Alzheimer’s disease (AD)?

**What is the bottom line?**

- Mendelian randomisation allows the use of huge sample populations and determines causality of the exposure outcome relationship. Using this approach, we found no good evidence that lung function and liability to COPD effects risk of AD.

**Why read on?**

- AD is the most common type of dementia; reports of a potential link between COPD and AD were first described nearly 30 years ago. If lung function and COPD have a causal effect on risk of AD, then they could represent modifiable risk factors.

## Introduction

Chronic obstructive pulmonary disease (COPD) is a disease of multimorbidity.^1^ In COPD, the presence of multimorbidity is associated with higher mortality, worse quality of life and increased healthcare utilisation.^2 3^ Impaired lung function measures such as forced expiratory volume in one second (FEV_1_) and forced vital capacity (FVC) have been found to be strongly associated with multimorbidity.^4^ However, it remains unclear if these multimorbidities are causally linked to lung function and disease, for example, through a proposed inflammatory overspill, or if they are due to shared risk factors, such as smoking.^5^ Therapeutic targets may be identified if specific causal mechanisms could be established.

Cognitive impairment is a common co-morbidity in COPD, with reported prevalence ranging from 10% to 61% and around 25% of older adults with dementia also have COPD.^6^ Cognitive impairment in COPD is associated with greater disability,^7^ poorer medication compliance,^8^ and risk of exacerbation and mortality.^7^ Poor pulmonary function in early life has been associated with increased odds of dementia later in life, even after adjustment for smoking.^9^

Alzheimer’s disease (AD) is the most common type of dementia^10^; its association with COPD is less well defined than general cognitive ability, but reports of a potential link between COPD and AD was first described nearly 30 years ago.^11^ Large retrospective observational case-control cohorts have reported increased risk of AD in patients with both COPD and reduced lung function.^12 13^ For example, Lutsey *et al* reviewed hospitalisation codes in the Atherosclerosis Risk In Communities Study for AD-related outcomes and reported that an OR of 1.24 for AD-type dementia or mild cognitive impairment (MCI) in patients with COPD and OR 1.79 for those with a restrictive impairment compared with controls.^13^ If the lung function and COPD have a causal effect on risk of AD, then they could be modifiable risk factors.

Mendelian randomisation (MR) is an established genetic epidemiological method which can overcome problems of unmeasured confounders and reverse causation, typical of conventional observational epidemiology.^14^ MR allows causal inference through the use of genetic variants as proxies for non-genetic (modifiable) risk factors or health outcomes.^14^ MR uses genetic data, for example, single nucleotide polymorphisms (SNPs) that are associated with an exposure (in this case diagnosis of COPD or lung function), and uses them as instrumental variables to assess the causal effect of the exposure on the outcome of interest (in this case AD).^15^

Our objective was to use MR to investigate if there is any evidence of a causal effect between the exposures, lung function and liability to COPD and the outcome, AD.

## Methods

### Lung function

We used data from Shrine *et al* the largest currently available lung function genome wide association study (GWAS), n=400 102 which reported 279 genome wide significant SNPs (p<5×10^−9^).^16^ Lung function measurements used were FEV in 1 s (FEV_1_), FVC, FEV_1_/FVC ratio and peak expiratory flow (PEF). 140 of the SNPs were previously reported and explained 5.0%, 3.4%, 9.2% and 4.5% of the estimated heritability of FEV_1_, FVC, FEV_1_/FVC and PEF, respectively. The 139 new signals reported explained an additional 4.3%, 3.3%, 3.9% and 3.3% of the estimated heritability, respectively. A weighted risk score was associated with risk of COPD (p=6.64×10^-63^), with an OR of 1.55 for each SD of the risk score.^16 17^ Further details of the study population can be found in the online supplemental information and the reference.^16^

10.1136/bmjresp-2020-000759.supp12 Supplementary data

### Chronic obstructive pulmonary disease

We used 82 SNPs associated with COPD, as identified in Sarkonsakaplat *et al* case control GWAS,^18^ n=35 735 cases and 222 076 controls discovered in meta-analysis of 25 studies. COPD was defined by Global Initiative for Chronic Obstructive Lung Disease criteria; FEV_1_/FVC<0.7 and FEV_1_ <80% predicted. SNP’s discovered explained up to 7% of phenotypic variance. Further details of study population can be found in the online supplemental information and the reference.^18^

10.1136/bmjresp-2020-000759.supp13 Supplementary data

Eighty per cent and 77% of the Shrine *et al* and Sarkonsakaplat *et al* GWAS sample, respectively, were from the UK Biobank.^19^ In brief, the UK Biobank is a large prospective cohort study where>500 000 participants were recruited from 2006 to 2010 in the UK (54% female). Prebronchodilation lung function testing was performed by trained healthcare staff.

### Alzheimer’s disease

We used data from a meta-analysis of the International Genomics of Alzheimer’s disease (IGAP) consortium,^20^ Alzheimer’s Disease Sequencing Project (ADSP),^21^ and Psychiatric Genomics Consortium totalling 24 807 AD cases and 55 058 controls.^22 23^ All cases had clinical diagnoses of AD. Some participants of the ADSP cohort were previously also included in IGAP, so ADSP individuals that were duplicates based on the comparison of individual level genetic data between IGAP and ADSP were excluded.

There was no sample overlap between the exposure and outcome samples. All participants were of European ancestry.

### Statistical analysis

Statistical analysis was done using R Studio V.3.5.1. and the MRCIEU/TwoSampleMR R package.^24^

For all exposures SNPs LD-clumping was performed using European reference population and the ieugwasr:ld_clump tool (kb=10 000, r^2^ 0.001). Palindromic SNPs (ie, A/T and C/G SNPs) with intermediate allele frequencies were excluded. The remaining SNPs were harmonised.^25^ Steiger filtering was performed to remove variants that caused more variance of the outcome than the exposure, see supplementary material for more details.^26^ F-statistics of the SNPs used in analysis were calculated (F statistic = beta^2^/SE^2^). The higher the F-statistic the lower the chance of weak instrument bias.^27^

### Main MR analysis

Inverse variance weighting (IVW) was used for main effect estimate. This is a weighted regression of SNP-outcome on SNP-exposure associations combined where the y intercept is constrained to zero.

#### Assumptions and sensitivity analysis

MR assumes that the SNPs are strongly associated with the exposure. This can be directly tested in the discovery GWAS by checking the proportion of variance explained by the SNPs, and when performing MR by ensuring the F-statistic of the SNPs is >10.^27^ MR assumes that the SNPs only affect the outcome via the exposure, not via a confounder or due to a direct effect on the outcome. This is not directly testable, but we perform a number of sensitivity tests to reduce this risk. For full details of assumptions and sensitivity tests, see online supplemental material, a synopsis is provided here. Steiger filtering removes SNPs that explain more variance in the outcome than the exposure (which, if present, is likely via a confounder or direct effect). To account for the possibility of horizontal pleiotropy (IVs influence exposure and outcome through independent pathways), we performed MR Egger. To minimise the effect of unbalanced instruments on an overall estimate of the mean, weighted median and mode MR methods were performed. To assess for horizontal pleiotropy a funnel plot was made by plotting the effect against its precision (beta against SE). To ensure the results were not due to outliers with a large effect, a leave-one-out analysis was performed by re-estimating the total effect after sequentially excluding one SNP at a time and a single-SNP analysis, where the effect of each SNP was individually assessed via IVW analysis and represented in a forest plot.

Heterogeneity (the variability in causal estimates obtained for each SNP) is an indication of potential violation of assumptions. This was calculated and assessed with a Q statistic.

### Patient and public involvement

This study used only pre-existing data from cohort trials. Details of patient and public involvement in the UK Biobank are available.^19^ Several patient organisations are part of the governance boards of the individual consortia that are part of IGAP. No patients were directly involved in formulating the research question, design or analysis of this study. No patients were asked to advise on interpretation of the results. There are no specific plans to disseminate the results of the research to study participants, but the UK Biobank disseminates key findings from projects on its website.

## Results

After clumping, extracting SNPs from outcome GWAS, Steiger filtering and removal of palindromic SNPs, 131 SNPs were available for analysis. F-statistic for lung function GWAS exposures were: All traits=114, FEV_1_=72, FVC=75, FEV_1_/FVC=150, making weak instrument bias unlikely.^16^

We found minimal evidence for a causal effect of lung function (all traits) on AD, (IVW OR (OR):1.02 per SD; 95% CI: 0.91 to 1.13; p value 0.68). This result was further confirmed in a sensitivity analysis using both weighted median (OR:1.01 per SD; 95% CI 0.86 to 1.19, p =0.81), and weighted mode MR (OR 0.99 per SD;95% CI 0.78 to 1.19), p=0.81). risk of AD. The MR-Egger causal estimation produced similar results with an OR 1.05 per SD (95% CI 0.79 to 1.34; p 0.71). The CI of the MR-Egger is wider than that of IVW, consistent with the lower statistical power of this test.

**Figure 1:**
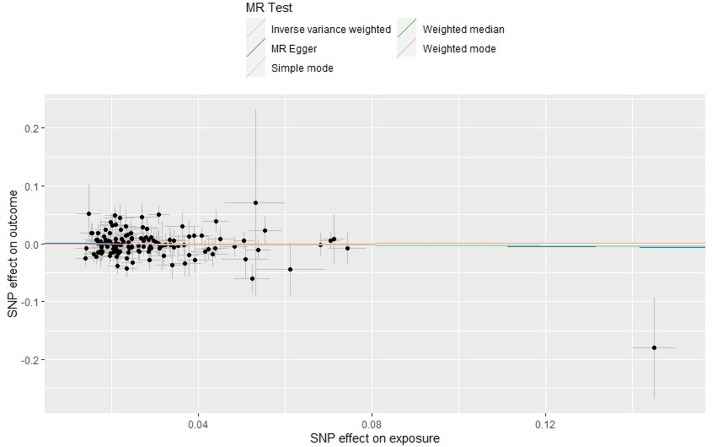
Scatter plot of the single nucleotide polymorphism (SNP) effect on lung function trait and SNP effect on Alzheimer’s disease. Each point on the graph represents the SNP-outcome association plotted against the SNP-exposure association. Bars indicate 95% CIs. Coloured lines represent analysis method used. This shows no effect of lung function on Alzheimer’s disease. Mendelian randomisation (MR) Egger intercept is close to zero indicating no unbalanced directional pleiotropy.

Figure 1 plots each individual SNP-exposure effect against SNP outcome with the coloured lines representing each statistical test. Increasing lung function (exposure) does not have a consistent effect on AD (outcome).

Table 1 shows that these results were consistent when analysing lung function traits FEV_1_, FVC, and FEV_1_/FVC individually with little evidence of a causal association on AD with confidence intervals crossing one for all statistical tests.

**Table 1:** Two-sample MR results of lung function traits^16^ on Alzheimer’s disease^22^

		Lung function trait (exposure)			
		FEV_1_, FVC, FEV_1_/FVC, PEF	FEV_1_	FVC	FEV_1_/FVC
No. SNPs used		131	42	46	73
IVW	OR per SD	1.02	1.04	1.08	0.99
	95% CI	0.91 to 1.13	0.82 to 1.32	0.85 to 1.37	0.88 to 1.13
	P value	0.68	0.73	0.51	0.97
	Q_p-value*	0.26	0.30	0.19	0.71
Weighted median	OR per SD	1.01	1.15	1.14	0.95
	95% CI	0.86 to 1.19	0.82 to 1.61	0.83 to 1.58	0.79 to 1.15
	P value	0.81	0.39	0.39	0.62
Weighted mode	OR per SD	0.99	1.07	1.04	0.97
	95% CI	0.78 to 1.26	0.60 to 1.90	0.61 to 1.78	0.74 to 1.26
	P value	0.97	0.80	0.86	0.84
MR Egger	OR per SD	1.05	1.22	0.97	0.95
	95% CI	0.79 to 1.34	0.57 to 2.59	0.36 to 2.62	0.69 to 1.31
	P value	0.71	0.59	0.96	0.77

*A test for heterogenity. If this was <0.05 it would suggest heterogenity.

FEV, forced expiratory volume; FVC, forced expiratory volume; IVW, inverse variance weighting; MR, Mendelian randomisation; PEF, peak expiratory flow; SNPs, single nucleotide polymorphisms.

**Figure 2:**
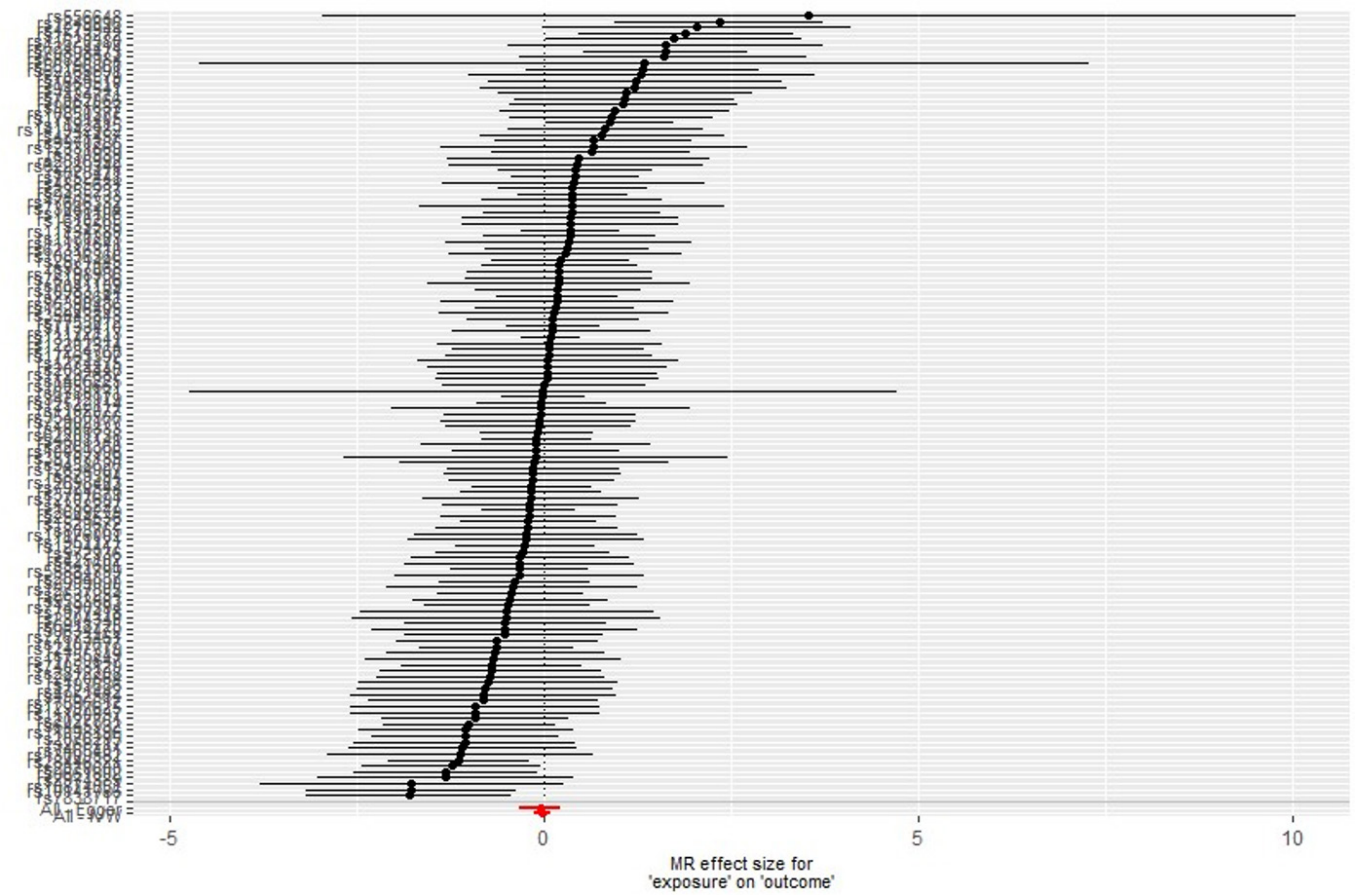
Single single nucleotide polymorphism (SNP) analysis of lung function traits on Alzheimer’s disease. Each point represents individual SNP calculated effect size for lung function on the odds of Alzheimer’s disease. Bars indicate 95% CI. MR, Mendelian randomisation.

We used single-SNP analyses to determine the effect of each lung function SNP on the odds of AD (figure 2). The SNP rs2070600 may be an outlier due to its comparatively large effect on both lung function and AD. Polymorphisms in this SNP have been described as having a weak effect on AD risk.^28^ However, despite excluding this SNP from the analysis, the results were similar (eg, see leave-one-out analysis in online supplemental figure E1).

10.1136/bmjresp-2020-000759.supp1 Supplementary data

Each SNP beta was plotted against its inverse standard error (online supplemental figure E2) producing a funnel shape indicating no heterogeneity. In addition to these visual tests, we found little evidence of heterogeneity using a Q statistic when lung function traits were combined or assessed individually (table 1. Q_p value>0.51). MR-Egger intercept was <0.001, visually displayed in figure 1, indicating there was no unbalanced horizontal pleiotropy.

10.1136/bmjresp-2020-000759.supp2 Supplementary data

After clumping, extracting SNPs from outcome GWAS, Steiger filtering and removal of palindromic SNPs, 53 SNPs for liability to COPD were available for analysis in the Alzheimer’s outcome GWAS. The remaining SNPs had an F-statistic of 54, making weak instrument bias unlikely. Results are displayed in table 2.

**Table 2:** Two-sample Mendelian randomisation (MR) results of chronic obstructive pulmonary disease (COPD)^18^ on Alzheimer’s disease^42^

	COPD	
No. SNPs used		53
IVW	OR per SD (95% CI)	0.97 (0.92 to 1.03)
	P value	0.40
	Q_p-value	0.57
Weighted Median	OR per SD (95% CI)	0.97 (0.90 to 1.05)
	P value	0.52
Weighted mode	OR per SD (95% CI)	0.96 (0.86 to 1.08)
	P value	0.56
MR-Egger	OR per SD (95% CI)	1.10 (0.93 to 1.31)
	P value	0.23

IVW, inverse variance weighting.

We found minimal evidence for an effect of liability to COPD on risk of AD (IVW OR: 0.97 per SD; 95% CI 0.92 to 1.03; p 0.40). This result was further confirmed in our sensitivity analysis using both weighted median (OR: 0.97 per SD; 95% CI 0.90 to 1.05; p=0.52), and weighted mode MR (OR: 0.96 per SD; 95% CI 0.86 to 1.08; p=0.56). The MR-Egger causal estimation produced an OR 1.11 per SD (95% CI 0.93 to 1.31; p 0.2), the only test to show a direction of effect of increasing COPD causing increased risk of AD. Online supplemental figures E3–6 are available in online supplemental information, demonstrating that results were not driven by an individual SNP. There was no evidence of heterogeneity, with a Q-pvalue 0.57. Flow charts of analysis path are available in supplement (online supplemental figures E7–11, online supplemental Appendix 5 5). A spreadsheet detailing SNPs used in final analyses is available as online supplemental information.

10.1136/bmjresp-2020-000759.supp3 Supplementary data

10.1136/bmjresp-2020-000759.supp4 Supplementary data

10.1136/bmjresp-2020-000759.supp5 Supplementary data

10.1136/bmjresp-2020-000759.supp6 Supplementary data

10.1136/bmjresp-2020-000759.supp7 Supplementary data

10.1136/bmjresp-2020-000759.supp8 Supplementary data

10.1136/bmjresp-2020-000759.supp9 Supplementary data

10.1136/bmjresp-2020-000759.supp10 Supplementary data

10.1136/bmjresp-2020-000759.supp11 Supplementary data

## Discussion

### Evidence before this study

Our results indicate that there is minimal evidence of a causal association between lung function or liability to COPD and risk of AD. This is in contrast to two large observational studies,^12 13^ which do report an association between COPD and AD. The observed associations may be due to unmeasured confounding by risk factors common to both COPD and AD such as smoking, physical inactivity, social deprivation and lower educational attainment.^29^ The observational studies may have inadvertently included other forms of dementia other than AD, for example vascular dementia resulting from cerebrovascular or neurological damage. Apolipoprotein e4 allele is the biggest risk factor for AD whereas it is thought that COPD affects cognition via vascular effects. There is evidence that COPD and reduced lung function is associated with micro and macrovascular damage that could mediate the relationship.^30–32^ It is possible that vascular dementia is causally linked to COPD and lung function, but this outcome was not included in our analysis which was restricted to AD only. Cognitive dysfunction and MCI are well described in COPD.^6^ It may be that this association is causal, but that patients do not progress to AD due to their lung disease. Survivor bias (where selection is conditional on survival to recruitment) can be of concern in studies involving potentially fatal diseases of later life.^33^ Potentially, patients with COPD would be less likely to be recruited to a GWAS, biasing the MR towards a null. Observational studies performed by analysing health records may be less likely to be affected by this.

### Impact of this study

This analysis uses two-sample MR to explore a causal association between lung function, COPD and AD. The increasing incidence of AD in Western society has been described as an epidemic.^34^ COPD is responsible for 5% of global disability-adjusted life years and 5% of total deaths.^35^ Consequently, prevention and treatment of both COPD and AD is a global health priority. Although there have been efforts to search for causal mechanisms linking the two diseases, our analysis using multiple means of assessing causation would suggest scientific attention and health prevention resources may be better focused on overlapping risk factors such as smoking, diet and physical activity,^36 37^ rather than attempts to reduce risk of AD by improving lung function or reducing liability to COPD alone.

### Strengths and limitations

MR has multiple advantages. Genetic variants are not influenced by behavioural or environmental factors minimising reverse causality (where the outcome, or early stages of the disease process that leads to the outcome, influences the exposure).^20^ Additionally, the effects are equivalent to lifetime differences, reducing issues relating to transient fluctuations.

By using randomly assigned genetic variants as an exposure, two-sample MR methodology eliminates many confounders in observational epidemiology.^14^ We used a large number of lung function and COPD SNPs, that reached genome wide significance in large samples.^16 18^ Other genetic variants that do not reach genome wide significance will contribute to complex traits, such as lung function and COPD, but were not included.^38^

It is important to ensure that the assumptions of MR are met when dealing with SNPs for complex phenotypes like lung function and COPD. We adhered to proposed methodological guidelines of MR (Strengthening the Reporting of Observational Studiesin Epidemiology) which are designed to increase reliability of MR reporting.^39^ None of the sensitivity tests provided strong evidence for a violation of the MR assumptions. The 15q25 locus is known to have strong associations with smoking behaviour, which could bias our results.^40^ When reviewing the SNPs for in our two-sample MR analysis, only 6 of the 279 SNPs are in chromosome 15, and none of are in the region of concern.^16^ When reviewing our COPD GWAS, only 4 of the 82 SNPs are in chromosome 15, and only one SNP from our COPD GWAS is in this locus (rs55676755).^18^ However, this SNP was not found in the outcome GWAS so was not included in our analysis. Therefore, none of the SNPs used were from the 15q25 locus. COPD is a binary trait, so our SNPs confer liability to COPD. As this is a Two Sample MR study, we do not know how many participants in the outcome population had COPD. COPD is a clinical diagnosis with set spirometric thresholds, whereas in the discovery GWAS a diagnosis of COPD was made based on spirometric criteria alone. This was done by dichotomising continuous traits. Dichotomisation of continuous traits in MR studies can make interpretation of the causal estimate less reliable, but MR can still be a valid test of the causal null hypothesis for a binary exposure.^41^

As the SNPs were discovered in populations of those with European ancestry, the results may not be generalisable to other populations. It is conceivable that there is a biological pathway that could cause lung function/COPD to have an effect of AD, but we do not have a SNP that affects such a pathway.

## Conclusions

Lung function and liability to COPD are not causally associated with an increased risk of AD. Previous observational studies showing and association between impaired lung function or COPD and AD are most likely due to unmeasured confounding.

## Data Availability

Data sharing not applicable as no datasets generated and/or analysed for this study. Data used was summary data freely available in supplementary tables or from corresponding authors of respective GWAS.
