# Supplementary material for: Examining the possible causal relationship between lung function, COPD and Alzheimer’s disease: a Mendelian randomisation study
Source: BMJ Open Respir Res. 2021 Jul 7;8(1):e000759. doi: 10.1136/bmjresp-2020-000759 (PMC8264898; doi:10.1136/bmjresp-2020-000759)
Supplement: Supplementary data [file bmjresp-2020-000759supp013.pdf]

SUPPLEMENTARY MATERIAL

Examining the possible causal relationship between Lung Function, COPD and Alzheimer's Disease. A Mendelian Randomization Study

Daniel H Higbee, Raquel Granell, Esther Walton, Roxanna Korologou-Linden, George Davey Smith, James W Dodd

Contents

Appendix 1. Figures for Lung function SNPs

Appendix 2. Figures for COPD specific SNPs

Appendix 3. Assumptions and tests

Appendix 4. Details of sample populations

Appendix 5. Flow chart of analyses

Appendix 6. Data availability and ethical approval

Appendix 1. Figures for Lung function SNPs

Figure E1. Leave-one-out analysis of lung function traits on Alzheimer's disease

Each point represents the IVW estimate if the SNP on the y axis was left out of total analysis. Bars indicate 95% confidence intervals, demostrating that no individual SNP is driving the causal effect estimate.

Figure E2. Funnel plot of heterogeneity of causal effects of lung function traits on Alzheimer's disease

Each point is a SNP with its beta plotted against its inverse standard error. As the graph is funnel shaped, it indicates no heterogeneity.

Appendix 2. Figures for COPD SNPs

Figure E3. Scatter Plot of IVW COPD and AD

Each point on the graph represents the SNP-outcome association plotted against the SNP-exposure association. Bars indicate 95% confidence intervals. Coloured lines represent analysis method used. This shows no signficant effect of COPD on Alzheimer's disease . MR Egger intercept is close to zero indicating no unbalanced directional pleiotropy.

Figure E4. Single SNP Analysis of COPD and AD

Each point represents individual SNP calculated effect size for COPD on odds of Alzheimer's disease . Bars indicate 95% CI.

Figure E5. Leave one out analysis of COPD and AD

Each point represents the IVW estimate if the SNP on the y axis was left out of total analysis. Bars indicate 95% confidence intervals. It demostrates that no individual SNP is driving the causal effect estimate.

Figure E6. Funnel Plot analysis of COPD and AD

Each point is a SNP with its beta plotted against its inverse standard error. As the graph is funnel shaped, it indicates no heterogeneity.

Appendix 3 Assumptions and tests

Assumptions

We assume that our IVs have a true association with the exposures. This has been rigorously statistically tested in the discovery GWAS and effect estimation.(1, 2) F-statistic calculation shows that all exposure SNPs were unlikely to be weak instruments. In both GWAS papers SNPs discovered are related to specific genes, cell types and biological pathways for lung tissue development.

We assume that our SNPs do not affect AD except via their effect on LF/COPD, and that the SNPs have no associations with any confounders that are also associated with AD. Although not possible to directly tests, our sensitivity and heterogeneity tests reduce the risk these assumptions were violated. To account for the possibility of horizontal pleiotropy (IVs affect multiple pathways) we performed MR Egger, weighted median and weighted mode tests. MR-Egger is similar to IVW except the y intercept is unconstrained. If the y intercept of the MR-Egger is not equal to zero then either there is unbalanced horizontal pleiotropy (the average pleiotropic effect differs from zero) or the pleiotropic effects are independent from the genetic association with the risk factor, or both.(3) Although power is lower compared to IVW, the gradient of the MR-Egger gives a causal estimate of the dose–response relationship between the genetic associations with the risk factor and those with the outcome, providing additional evidence for causal affect. To help avoid the effect of unbalanced instruments on an overall estimate of the mean by the IVW method, weighted median and mode MR methods were performed. A weighted median MR gives a consistent estimate of the causal effect when at least 50% of the weight comes from valid IVs, giving a greater robustness with strongly outlying causal estimates.(4) A weighted mode MR calculates an estimate based on the set of SNPs that form the largest homogenous cluster, which attempts to avoid the impact of invalid instruments.(5)

There was no evidence of population stratification (when subgroups within a sample are of different genetic ancestry) as assessed by linkage disequilibrium score regression in the original GWAS.

Steiger Filtering

Steiger filtering estimates each SNP's rsq.exposure and rsq.outcome in the outcome population.(6) Those SNPs that explain more variance in the outcome than exposure are excluded, as they could led to a reverse causal relationship. SNPs were removed if they explained more variance of the outcome than the exposure. SNPs were removed if they explained more variance of the outcome than the exposure. Necessary information to perform Steiger filtering includes knowing the case and control numbers for each SNP. In our main Alzheimer's meta-analysis outcome population, we only know this for the PGC cohort. Therefore, we assumed that for the SNPs tested for in the ADSP and IGAP cohorts, every SNP had the same case and control number as the overall participant numbers. For ADSP 4,343 cases and 3,165 controls, for IGAP 17,008 cases and 14,471 controls. A prevalence of the outcome as required for Steiger filtering, we stated that the prevalence of the outcome was 0.07. Similarly, we do not know the exact case and control number for each SNP in the COPD exposure. Therefore, we assumed that every SNP had the same case and control number as the total number of case (35,735) and control (222,076) participants in the study. We estimated the prevalence to be 0.1 as per the discovery GWAS. Lung function is a continuous trait, so does not require estimation of prevalence to perform Steiger filtering.

Appendix 4. Details of Sample Populations

Table E1. Description of GWAS samples used

Exposure	Data source (PMID)	Sample size (% cases)	% European	GWAS Adjustment
Lung function	UKBiobank & SpiroMeta	400,102	100	age, age^2, sex, height, smoking status
COPD	25 studies*	257,811 (13.8%)	100	age, age^2, sex, and height
**Outcome**	**Data source (PMID)**	**Sample size (% cases)**	**% European**	**GWAS Adjustment**
Alzheimer's Disease	IGAP, ADSP, PGC	79,865 (31%)	100	ADSP&PGC = gender, batch, 4 principal components<br>PGC = age

*Please see supplementary table 1 of reference[(2)] for full table naming each study with description of respective: cases/controls number, smoking status, age, FEV_1% and FEV_1/FVC

Appendix 5. Flow chart of analysis

Study protocols were not pre-registered.

Figure E7. Flow chart of analysis for all lung function trait SNPs

Figure E8. Flow chart of analysis for FEV_1 SNPs

Figure E9. Flow Chart of analysis for FVC SNP's

Figure E10. Flow Chart of analysis for effect FEV_1/FVC SNPs

Figure E11. Flow Chart of analysis for COPD liability SNPs (2)

Appendix 6. Data availability and ethical approval

Data used was summary data freely available in supplementary tables or from corresponding authors of respective GWAS. As we only used summary data that is freely available, we did not seek ethical approval for this study.

References

1. Shrine N, Guyatt AL, Erzurumluoglu AM, Jackson VE, Hobbs BD, Melbourne CA, et al. New genetic signals for lung function highlight pathways and chronic obstructive pulmonary disease associations across multiple ancestries. Nature Genetics. 2019;51(3):481-93.
2. Sakornsakolpat P, Prokopenko D, Lamontagne M, Reeve NF, Guyatt AL, Jackson VE, et al. Genetic landscape of chronic obstructive pulmonary disease identifies heterogeneous cell-type and phenotype associations. Nature Genetics. 2019;51(3):494-505.
3. Bowden J, Davey Smith G, Burgess S. Mendelian randomization with invalid instruments: effect estimation and bias detection through Egger regression. International journal of epidemiology. 2015;44(2):512-25.
4. Bowden J, Davey Smith G, Haycock PC, Burgess S. Consistent Estimation in Mendelian Randomization with Some Invalid Instruments Using a Weighted Median Estimator. 2016;40(4):304-14.
5. Hemani G, Bowden J, Davey Smith G. Evaluating the potential role of pleiotropy in Mendelian randomization studies. Human Molecular Genetics. 2018;27(R2):R195-R208.
6. Hemani G, Tilling K, Davey Smith G. Orienting the causal relationship between imprecisely measured traits using GWAS summary data. PLoS genetics. 2017;13(11):e1007081.
