# Supplementary figures and images for: Examining the possible causal relationship between lung function, COPD and Alzheimer’s disease: a Mendelian randomisation study
Source: BMJ Open Respir Res. 2021 Jul 7;8(1):e000759. doi: 10.1136/bmjresp-2020-000759 (PMC8264898; doi:10.1136/bmjresp-2020-000759)

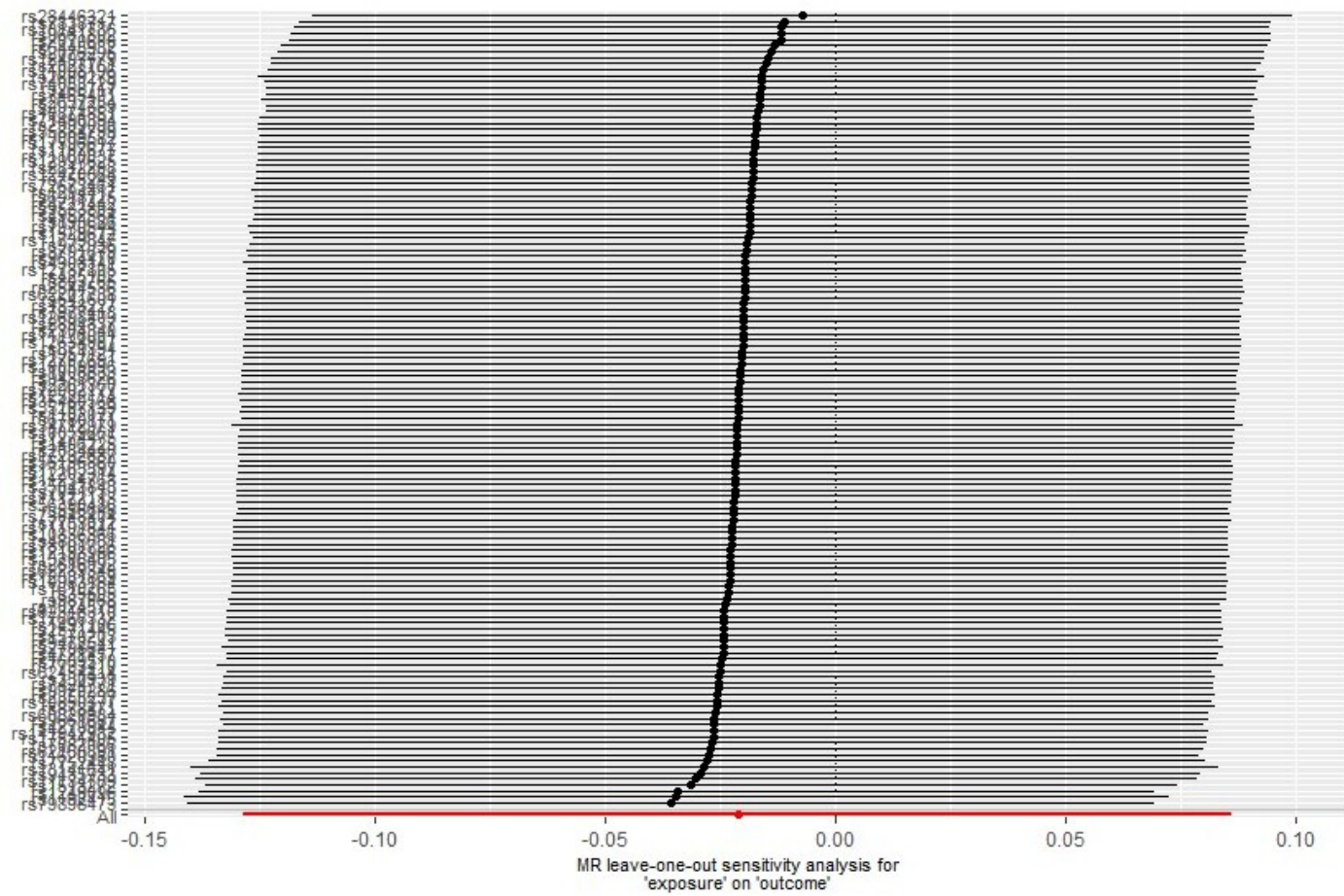

Supplement: Supplementary data [file bmjresp-2020-000759supp001.pdf]

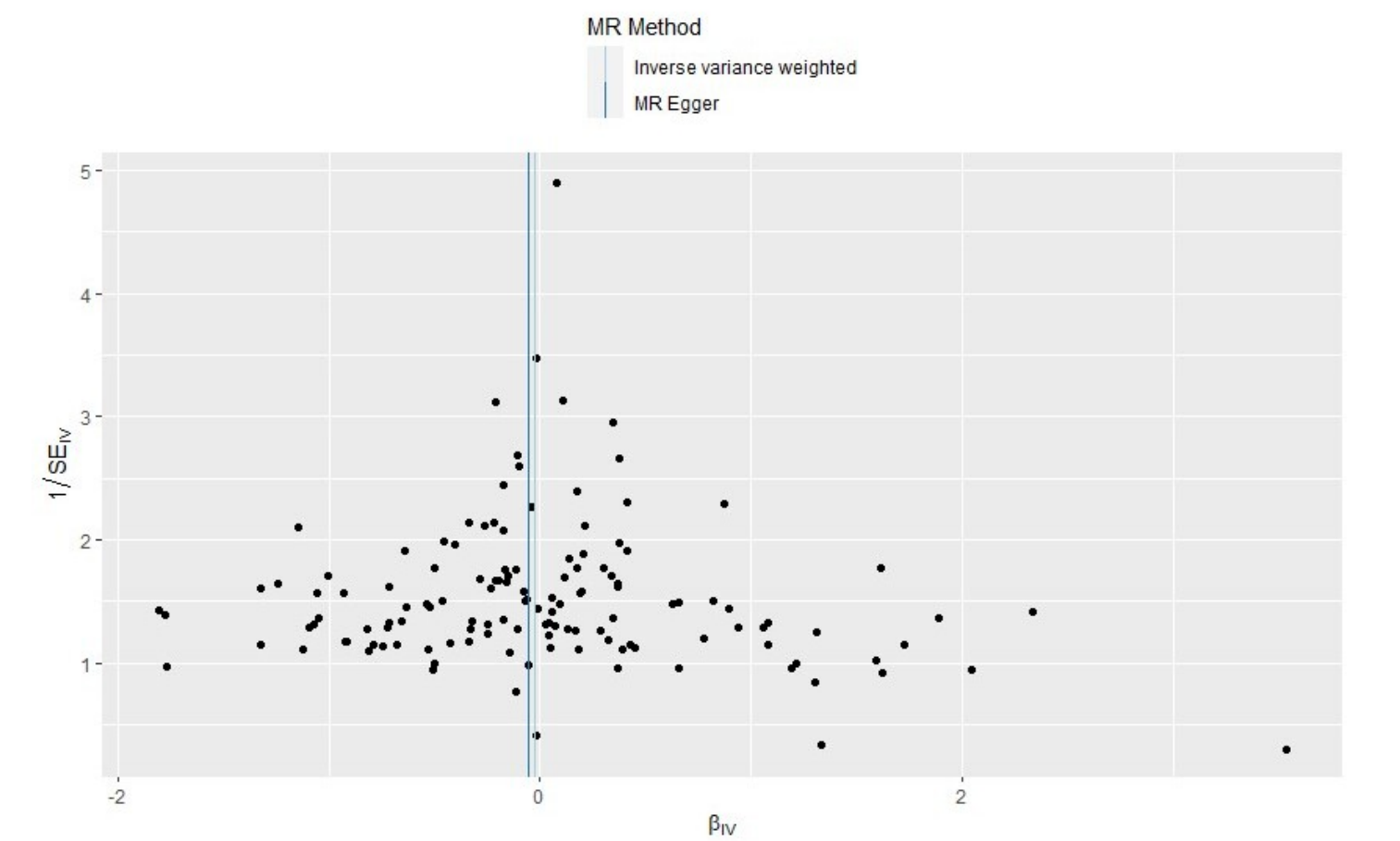

Supplement: Supplementary data [file bmjresp-2020-000759supp002.pdf]

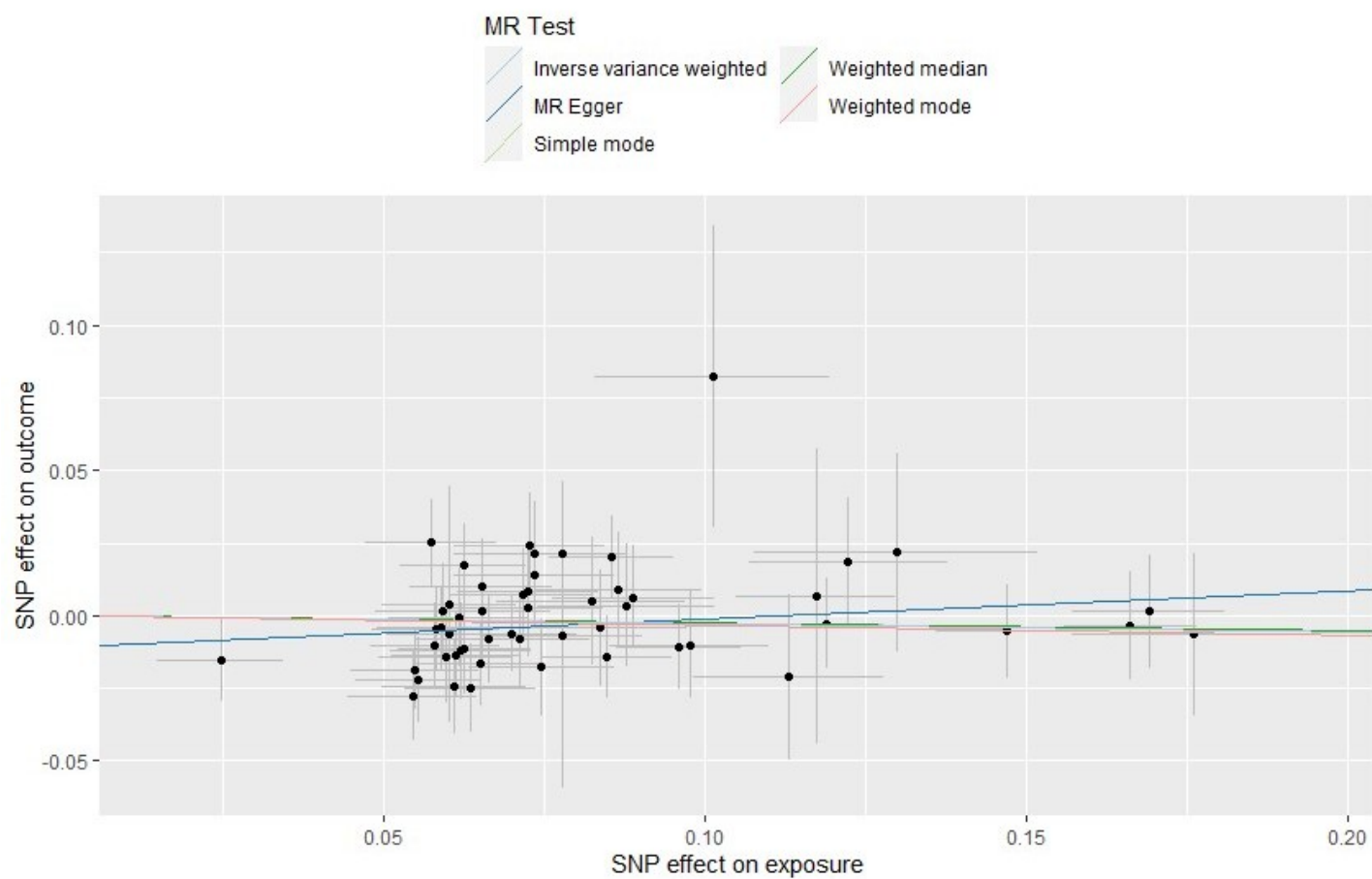

Supplement: Supplementary data [file bmjresp-2020-000759supp003.pdf]

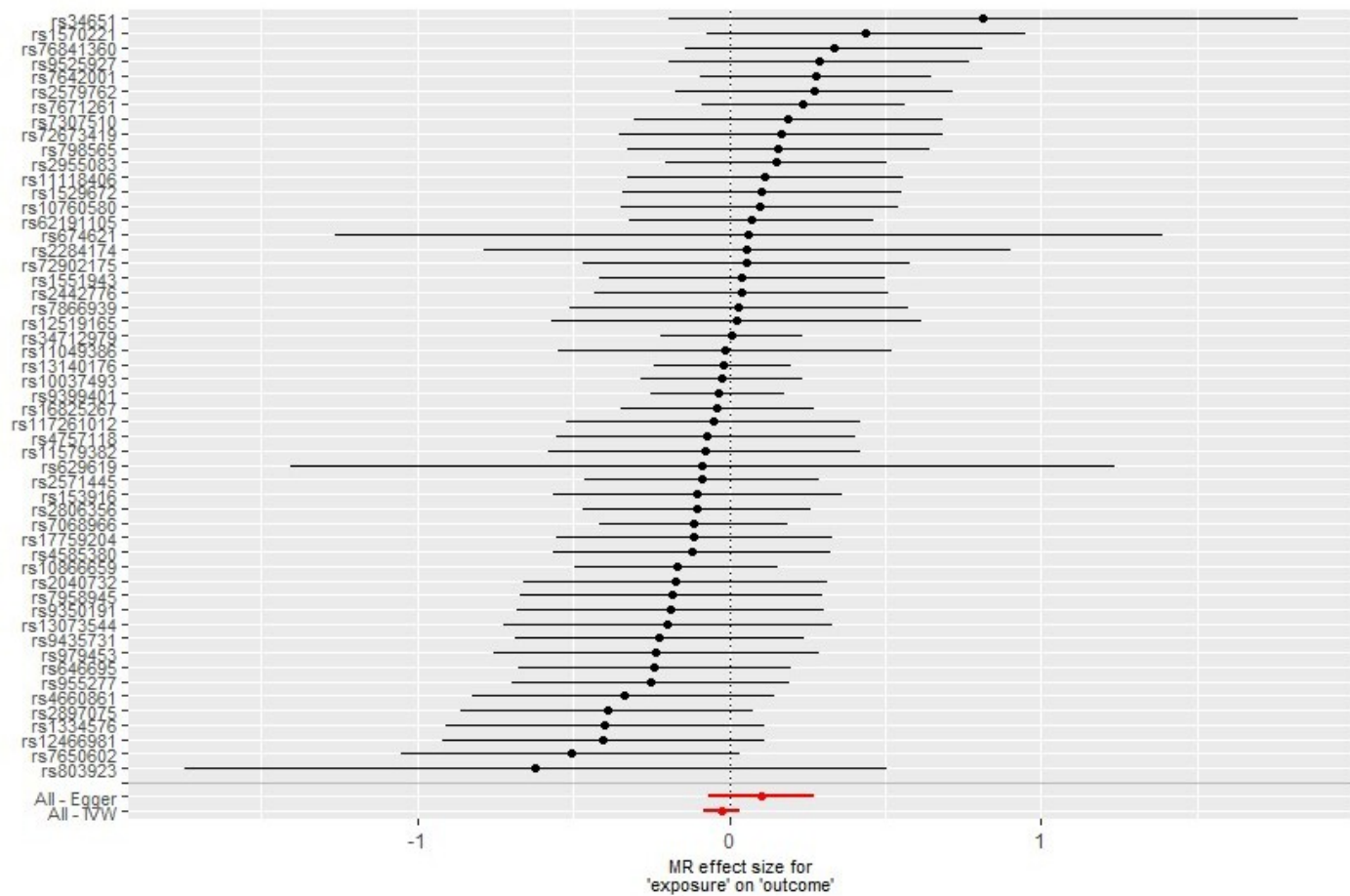

Supplement: Supplementary data [file bmjresp-2020-000759supp004.pdf]

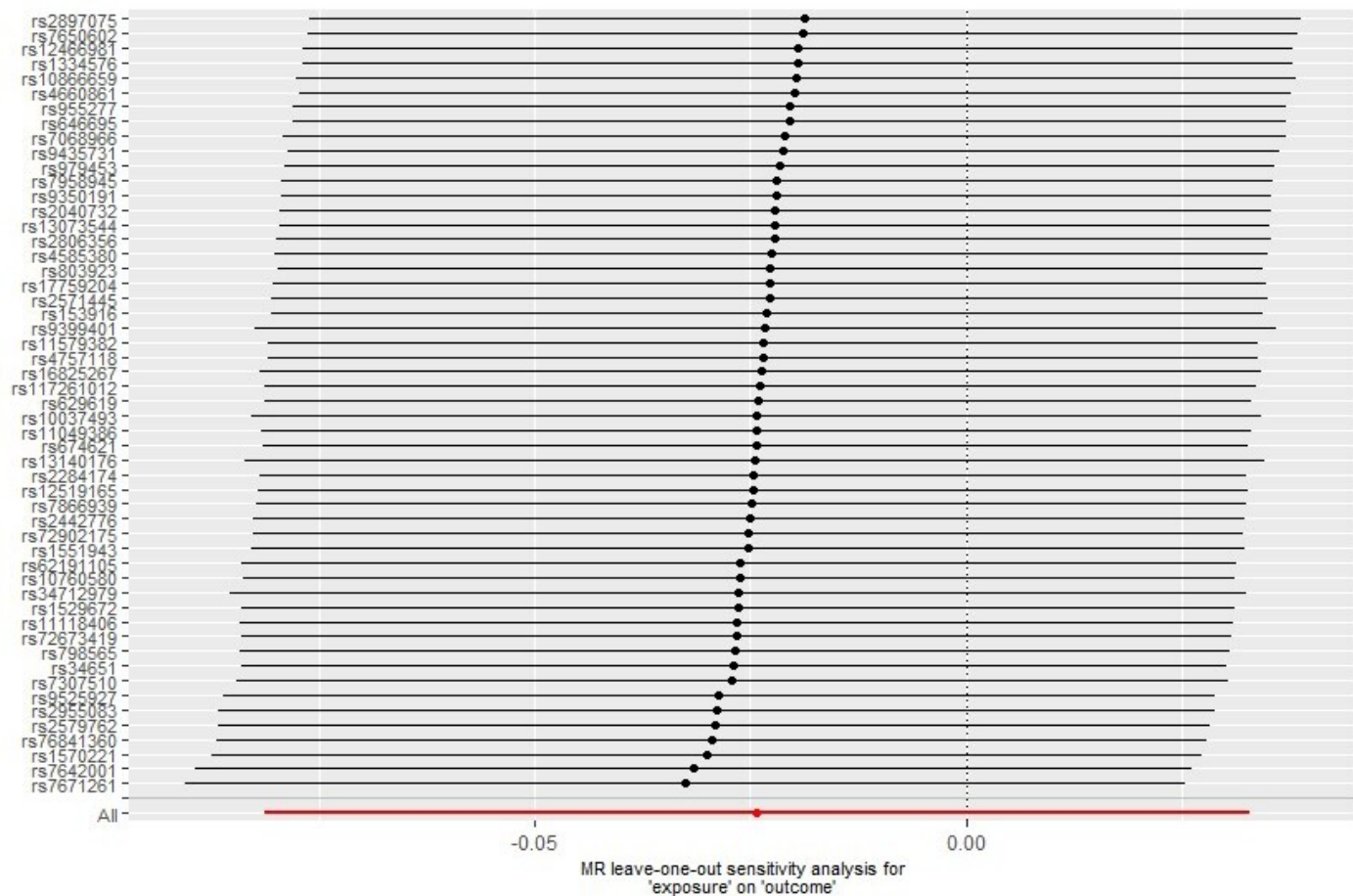

Supplement: Supplementary data [file bmjresp-2020-000759supp005.pdf]

MR Method

- Inverse variance weighted
- MR Egger

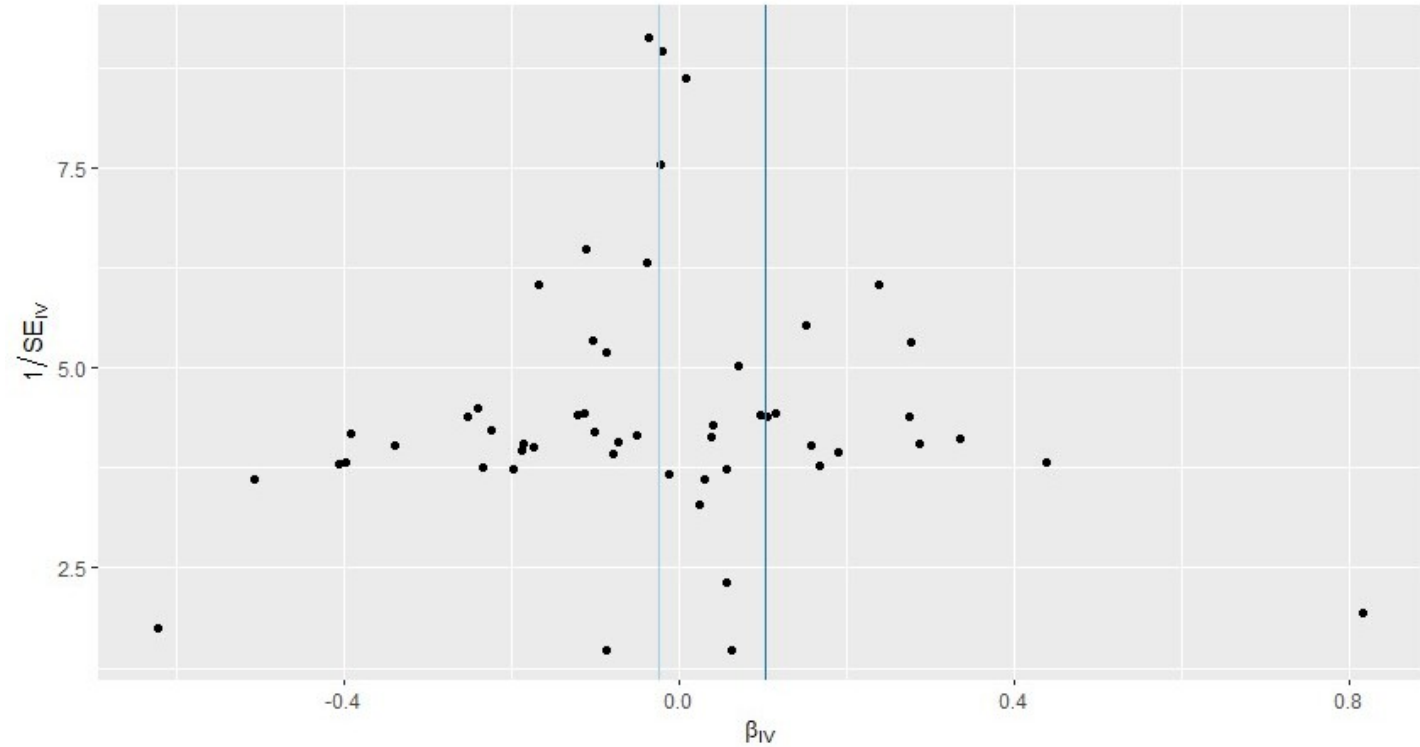

Supplement: Supplementary data [file bmjresp-2020-000759supp006.pdf]

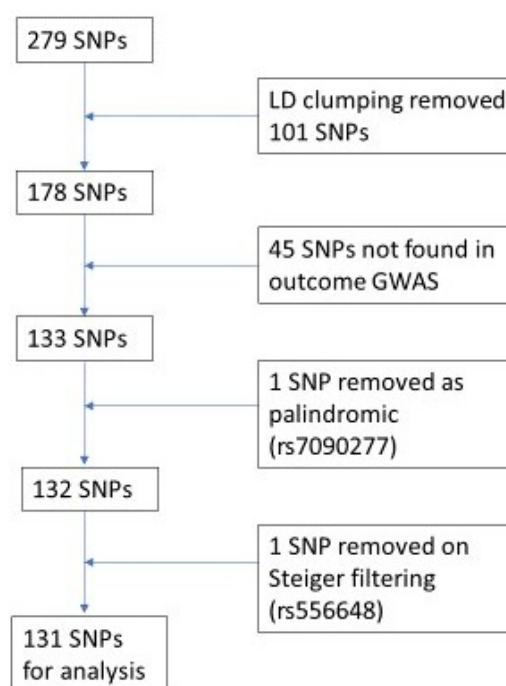

Supplement: Supplementary data [file bmjresp-2020-000759supp007.pdf]

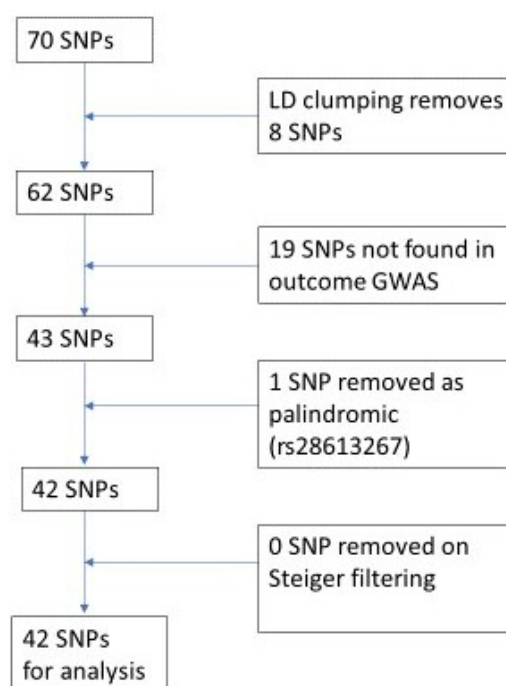

Supplement: Supplementary data [file bmjresp-2020-000759supp008.pdf]

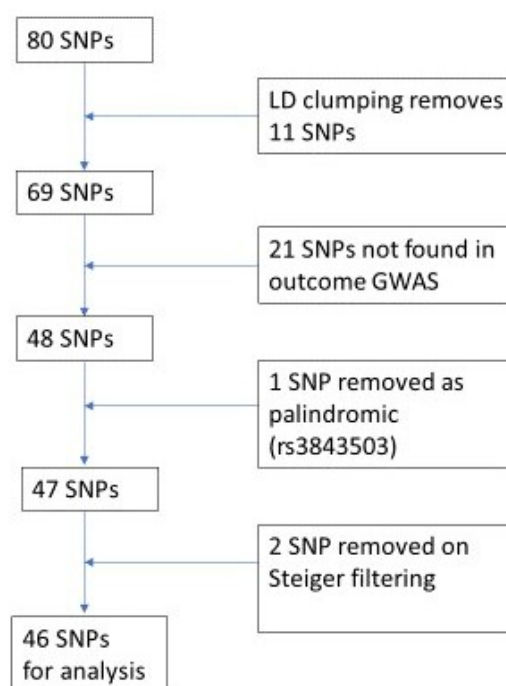

Supplement: Supplementary data [file bmjresp-2020-000759supp009.pdf]

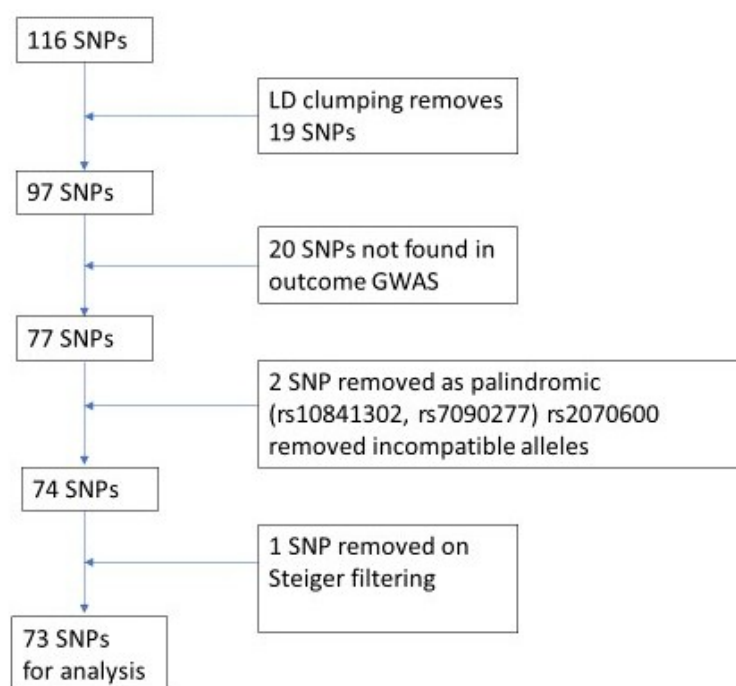

Supplement: Supplementary data [file bmjresp-2020-000759supp010.pdf]

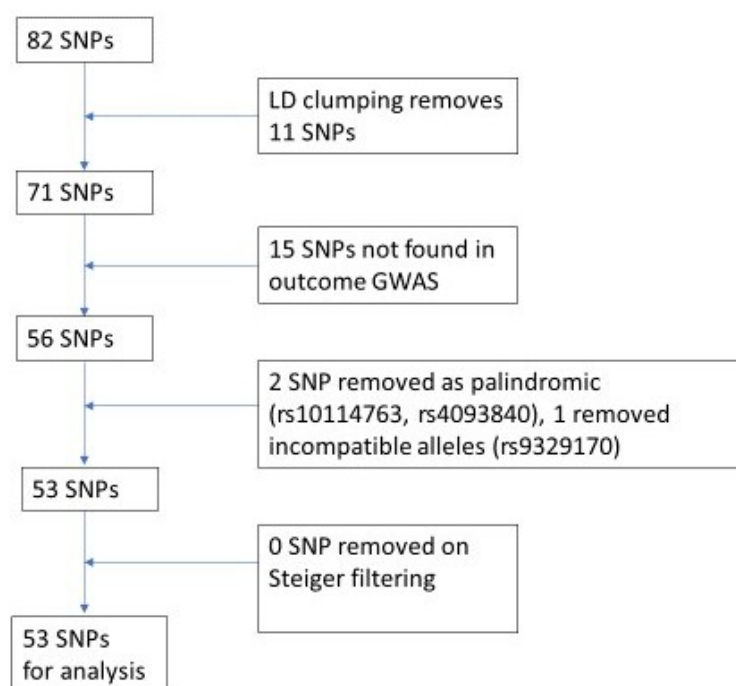

Supplement: Supplementary data [file bmjresp-2020-000759supp011.pdf]
